# Fluorescence in situ hybridization detection of chromosome 7 and/or 17 polysomy as a prognostic marker for cholangiocarcinoma

**DOI:** 10.1038/s41598-022-11945-8

**Published:** 2022-05-19

**Authors:** Raksawan Deenonpoe, Prakasit Sa-ngiamwibool, Sasithorn Watcharadetwittaya, Malinee Thanee, Kitti Intuyod, Thachanan Kongpan, Sureerat Padthaisong, Rungtiwa Nutalai, Yaovalux Chamgramol, Chawalit Pairojkul

**Affiliations:** 1 Department of Pathology, Faculty of Medicine, Khon Kaen University, 123 Mittraparp Road, Muang District, Khon Kaen, 40002 Thailand; 2 Cholangiocarcinoma Research Institute (CARI), Khon Kaen University, Khon Kaen, Thailand; 3 Faculty of Allied Health Sciences, Burapha University, Chonburi, 20131 Thailand; 4 Present Address: Nuffield Department of Medicine, Wellcome Trust Centre for Human Genetics, University of Oxford, Oxford, UK

**Keywords:** Cancer, Genetics, Molecular biology, Gastroenterology, Medical research, Molecular medicine, Oncology, Pathogenesis

## Abstract

Cholangiocarcinoma (CCA) is highly endemic in the Northeast Thailand. Recently, chromosome aberrations provided new insights into pathogenesis of CCA. Therefore, chromosome aberration might be used as a prognostic factor and therapeutic planning of this cancer. This aim of this study is to examine the correlation between an increase of chromosome 7 (C7) and/or 17 (C17) copy number variants (CNVs) with clinicopathological data and the overall survival time (OS) of CCA patients using fluorescence in situ hybridization (FISH) assays. C7 and C17 CNVs were examined using FISH form 157 formalin-fixed paraffin-embedded (FFPE) tissues of CCA patients from Khon Kaen, Thailand between 2011 and 2015. OS was visualized using Kaplan–Meier plot. Univariate and multivariate analyses were used to determine the ability of the clinicopathological parameters to predict OS. C17 > trisomy (odd ratio, 6.944, *P* < *0.001*), C7/17 trisomy (odd ratio; 4.488, *P* = *0.019*), and C7/17 > trisomy (odd ratio; 6.723, *P* < *0.001*) were independently predictive factors for lymph node metastasis. Interestingly, an increase of C7, C17, and C7/17 CNVs in both trisomy and > trisomy was independently correlated with short median OS. An increased of C7 and/or 17 have a potential as a poor prognostic marker in CCA patients.

## Introduction

Cholangiocarcinoma (CCA) is a devastating cancer originated from epithelial cells of the bile duct located either intrahepatic (iCCA) or extrahepatic (eCCA) biliary tree, and the latter is further classified into perihilar (pCCA) or distal (dCCA) bile duct types^1^. CCA is a rare cancer because of the relatively low incidence worldwide. Nevertheless, the worldwide incidence of CCA has been increased over past decades^2^ especially in the Northeastern Thailand where has the highest incidence of CCA in the world^3,4^. In Thailand, the annual incidence of CCA in the Northern region was 85 cases per 100,000 persons, whilst that of the Southern region was 5.7 cases per 100,000 persons^5^. Khon Kaen is a city in the Northerastern Thailand, with a population of 1.7 million people, and an endemic area of the liver fluke *O. viverrini*, where CCA is highly prevalent with an age-standardized incidence rate (ASR) of 58.8 and 23.6 per 100,000 males and females, respectively^4^.

This variation of CCA incidence reflects the differences of risk factors for CCA in Thailand^1,5^. Although the definite causes of CCA are unclear, several factors, both infectious and non-infectious agents, have been proposed as risk factors for CCA development^1,6,7^. In the Northeastern Thailand, based on epidemiological and experimental studies, infection with small human liver fluke *Opisthorchis viverrini* has been recognized as the most important risk factor for CCA development^6,7^.

CCA is a heterogeneous group of malignancies arising from hepatic progenitor cells, biliary epithelial cells, or peribiliary glands (PBGs) of the intrahepatic and extrahepatic bile ducts^8–10^. However, intrahepatic CCA may also arise from transactivated hepatocytes^11,12^. In 2010, the American Joint Committee on Cancer reclassified CCAs into intrahepatic CCA (iCCA) and extrahepatic CCA (eCCA), using hepatic ducts as the separation point. The latter (eCCA) is further subdivided into perihilar CCA (pCCA) and distal CCA (dCCA) at the level of the cystic duct^10,13,14^. iCCAs are classified into mass-forming (MF), periductal-infiltrating (PI), and intraductal-growing (ID) types. The MF type is the most common type of iCCA. Histologically CCA is classified into papillary and tubular adenocarcinomas. The median survival time of the papillary type is 23.4 months, whereas that of the tubular type is 16.3 months^15^.

Because of the lack of early signs and specific biomarkers, CCA patients are often diagnosed at the late-stage leading to limited treatment choices. Accordingly, CCA patients usually have poor prognosis and short survival^16^. Prognostication is one of the important clinical and ethical approaches for clinicians involved in oncology and palliative care of all cancer types including CCA because it helps clinicians for planning the appropriate therapeutic strategy for advanced cancer patients^17^. Typically, prognostication is made based on several clinical factors but primarily based on tumor staging^18,19^. Apart from tumor staging, several factors have been reported as prognostic factors for advanced cancer patients^20^ including chromosomal aberration^21,22^.

Cancer is a multifactorial disease and is associated with multiple genetic abnormalities^22^. Chromosomal aberration is recognized as one of the most common genetic events in cancer development and progression^22–24^. Several types of chromosomal aberration of both structural and numerical abnormalities such as aneuploidy, polyploidy, translocation, deletion etc. have been identified in different cancer types^22^. Therefore, identification of chromosomal abnormalities in cancers can be clinically utilized, particularly cancer detection. In addition, a number of chromosomal aberrations are known to associate with several unfavorable clinical behaviors. Thus, some of chromosomal abnormalities have been used as prognostic factors for advanced cancer patients^21,22^. For CCA, several chromosomal aberrations such as 5q, 7p, 8q, 13q, 17q and 20q gains and 3p, 6q, 9p, and 17p losses have been reported^25–28^. These aberrations underlie the amplification of oncogenes such as epidermal growth factor receptor (EGFR: 7p12), epidermal growth factor receptor 2 (HER2: 17q22), platelet-derived growth factor subunit A (PDGFA: 7q22) or deletion of tumor suppressor genes particularly of cyclin-dependent kinase inhibitor 2A (CDKN2A), mucin 17 (MUC17) and tumor protein p53 (TP53) which are located on 9p21, 7q22.1 and 17p13, respectively^27,29,30^.

Fluorescence in situ hybridization (FISH) is one of the cytogenetic approaches that has been used to investigate chromosomal aberration in both clinical and research settings. Application of FISH technique on brushing smears can detect numerical and structural abnormalities of four chromosomes in patients with documented extrahepatic CCA^31^. For cancer cytogenetics, UroVysion^®^, a FISH-based diagnostic kit, has been approved by FDA for assisting in diagnosis of bladder cancer. This kit consists of specific probes to detect aneuploidy of chromosomes 3, 7 and 17, and also deletion of 9p21. Although the UroVysion^®^ was developed for diagnosis of bladder cancer, the usability of UroVysion^®^ assay in other cancer types has been studied including prognostication of CCA patients^32^. Correlation of the increase of chromosome 7 copy number with the shorter overall survival of CCA patients was demonstrated using UroVysion^®^ with the underscoring of the usefulness of UroVysion^®^ assay for prognostication of CCA patients^32^. Moreover, increase of the percentage of cells with gains of chromosomes 3, 7, and 17 and the percentage of polysomic cells was reported in biliary dysplasia and carcinoma compared with the benign tissue^33^.

However, the application of UroVysion^®^ assay as a prognostic marker for CCA patients in liver fluke endemic area in the Greater Mekong Subregion (GMS) including the Northeastern Thailand has not yet been demonstrated. It is known that genetic and epigenetic abnormalities of CCA patients in the GMS is different from those seen in CCA patients from other areas^34–36^. In this study, therefore, we focused on to detect polysomy of chromosomes 7 and 17 from the full range UroVysion^®^ assay. This is because previous studies suggested the association of polysomy of these 2 genes with poor prognosis of CCA and is relation to interesting oncogenes^8,37^. Then, we analysed the association of these chromosomal abnormalities with the survival rate of CCA patients. This study may provide an insight into the usefulness of preliminary probes of UroVysion® assay for prognostication of CCA patients especially those in liver fluke endemic area.

## Materials and methods

### Patients

A total of 157 formalin-fixed paraffin-embedded (FFPE) tissues of CCA patients who came to Srinagarind Hospital, Khon Kaen University, from 2011 to 2015 and underwent hepatectomy were obtained from the Pathology service unit, Department of Pathology, Khon Kaen University. The FFPE were finally diagnosed with pathologists after operation and confirmed by two experienced pathologists (P.S. and S.W.). Patients’ overall survival times were calculated since the surgical treatment date to the present, that is 26 November 2019. All of 157 FFPE specimens were collected from 157 CCA patients which consisted of 80 males and 77 females with the average age 30–70 years old. The informed consent was obtained from all subjects or their legal guardians. The study protocol was approved by the Ethics Committee of Khon Kaen University (No. HE621105, KKU 0301.6.2.9/564 dated 19 April 2019) and all methods were confirmed that were performed in accordance with relevant guidelines and regulations.

### Specimen preparation

Surgically resected specimens were processed by the standard formalin-fixed paraffin-embedding (FFPE) technique. The block of FFPE specimens were cut at 4 µm thickness for H&E staining. All H&E slides were marked the area of CCA by two experienced pathologists and then the slides were applied to construct tissue microarray (TMA) blocks. Tissue microarrays are compound paraffin blocks constructed by extracting cylindrical tissue core of 3 mm biopsies of marked area from all donor paraffin blocks which were re-embedded into a microarray or recipient block at defined array coordinates^38^. One slide of tissue microarray contains eight positive cases with CCA and one normal case control.

### Fluorescence in situ hybridization assay

FISH technique was performed on TMA slides of FFPE tissues. The TMA specimens were sectioned at 4 µm thickness. The slides were deparaffinized at 60 °C overnight then immersed into xylene ambient 5 min 3 times each after that dehydrated with 100% ethanol 2 times 1 min each. The slides were pretreated with saline sodium citrate (SSC) buffer at 80 °C for 30–45 min depends on the condition of tissue, then rinsed with purified water for 3 min. Then, the slides were soaked in 50 ml protease buffer with 75 mg protease at 37 °C for 30–45 min. The slides were dehydrated in an ascending series (70–100% (v/v)) ethanol. The FISH centromere enumeration probes (CEP) were specific for pericentromeric regions of chromosomes 7 (C7) and 17 (C17) (Abbott^®^, Laboratories Ltd; Illinois, USA). The probes preparation of one assay was mixed with hybridization buffer, probes and H_2_O, then, applied the probes mix to the target area on TMA slide, closed with coverslip and sealed with rubber cement. The hybridization step was performed on the ThermoBrite at 73 °C for 5 min followed by 37 °C 18 h. The first step of post-hybridization procedure, the slides were washed with 2X SSC/0.3% Tween 20 at ambient for 5 min and allowed coverslips to float off gently. Next, those slides were washed with 2X SSC/0.3% Tween 20 at 73 °C for 3 min. The last step, 4’,6-diammidino-2 phenylindole (DAPI) was counterstained, closed with coverslip and storaged at − 20 °C protected from the light. Those slides were investigated under a fluorescence microscope (Olympus BX63; Tokyo, JAPAN) using GenASis FISH View & Spot Counting (Applied Spectral Imaging; Yokneam Illit, ISRAEL).

### Evaluation criteria

**Figure 1:**
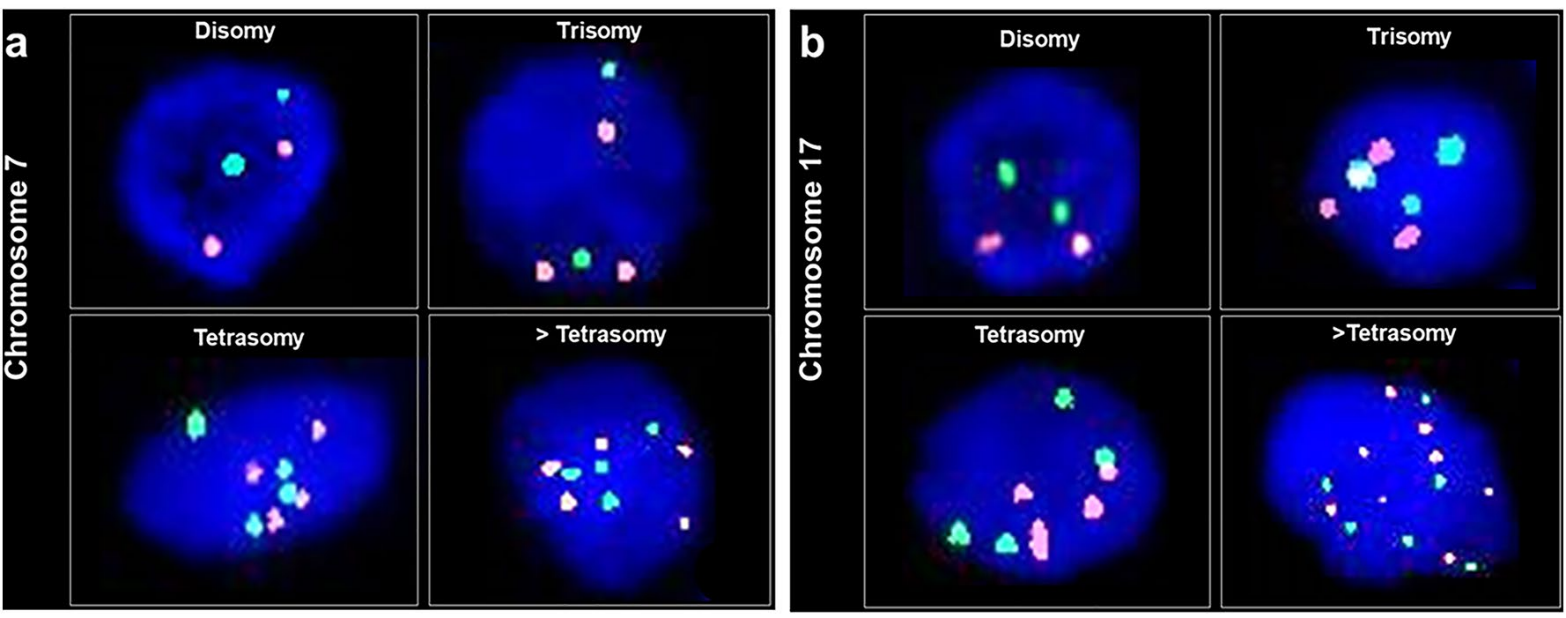
Representative FISH image (**a**,**b**). The pattern of chromosome 7 and 17 copy numbers including disomy (2 signals), trisomy (3 signals), tetrasomy (4 signals) and > tetrasomy (> 4 signals). Probes: CEP7; red, CEP17; green.

We analyzed cut-off values based on FISH signals from 100 nuclei from each 10 FFPE tissues of surgical samples from cholangitis patients (n = 10). The mean value of C7 (% X for non-disomy) (a gain of more than two chromosomes within a single cell) was 4.5%, the upper cut-off value (%) was 8%, which was the mean + two standard deviations. While, the mean value of C17 (% X for non-disomy) (a gain of more two chromosomes within a single cell) was 4%, with the upper cut-off value (%) of 6%. We analyzed 50 sequential, non-overlapping, well-visualized, epithelial cell nuclei and consecutive area in all samples under a fluorescence microscope (Olympus BX63; Tokyo, JAPAN). The numerical chromosome aberration phenotypes of hepatectomy specimens were identified as disomy, trisomy, tetrasomy and > tetrasomy upon an increase of chromosomes as shown in Fig. 1. According to few tetrasomy, then “ > trisomy” used as other representative group to statistical analysis. Polysomic cell was referred to an increase chromosome from those normal 2 CNVs. The number of counted cells was listed and the highest percentage of those phenotype was taken as the representative phenotype of individual patient. Each sample was independently examined by three investigators (RD, MT, and KI) who were unsighted to the clinicopathological data. If the assessments of the two investigators differed, a consensus was accomplished by discussion.

### Statistical analyses

Statistical analyses were carried out using the Statistical Package for the Social Sciences; SPSS software V.23.0 (IBM, Chicago, IL, USA). Comparison between signal copy numbers and the clinicopathological parameters of the CCA patients was determined using Pearson's χ2 or Fisher's Exact Test. A logistic regression model was used to evaluate the predictive ability with lymph node, gall bladder and distant organ metastasis. Following, a backward stepwise multinomial regression model was adjusted, and variables were kept in the model when *P* < 0.05. Overall survival (OS) was estimated by Kaplan–Meier analysis, and the curves were compared using log-rank tests. Regression analyses of survival data were conducted on OS defined as from the time of surgery to the time of death. Cox regression was used for univariate and multivariate analyses (backward stepwise model) of the ability of the clinicopathological parameters to predict OS. *P*-value of < 0.05 was considered to indicate statistical significance.

### Ethics approval

Ethics approval of the study was obtained from the Khon Kaen University Ethics Committee for Human Research No. HE621105 before experiment.

## Results

### Characteristics of CCA patients

**Figure 2:**
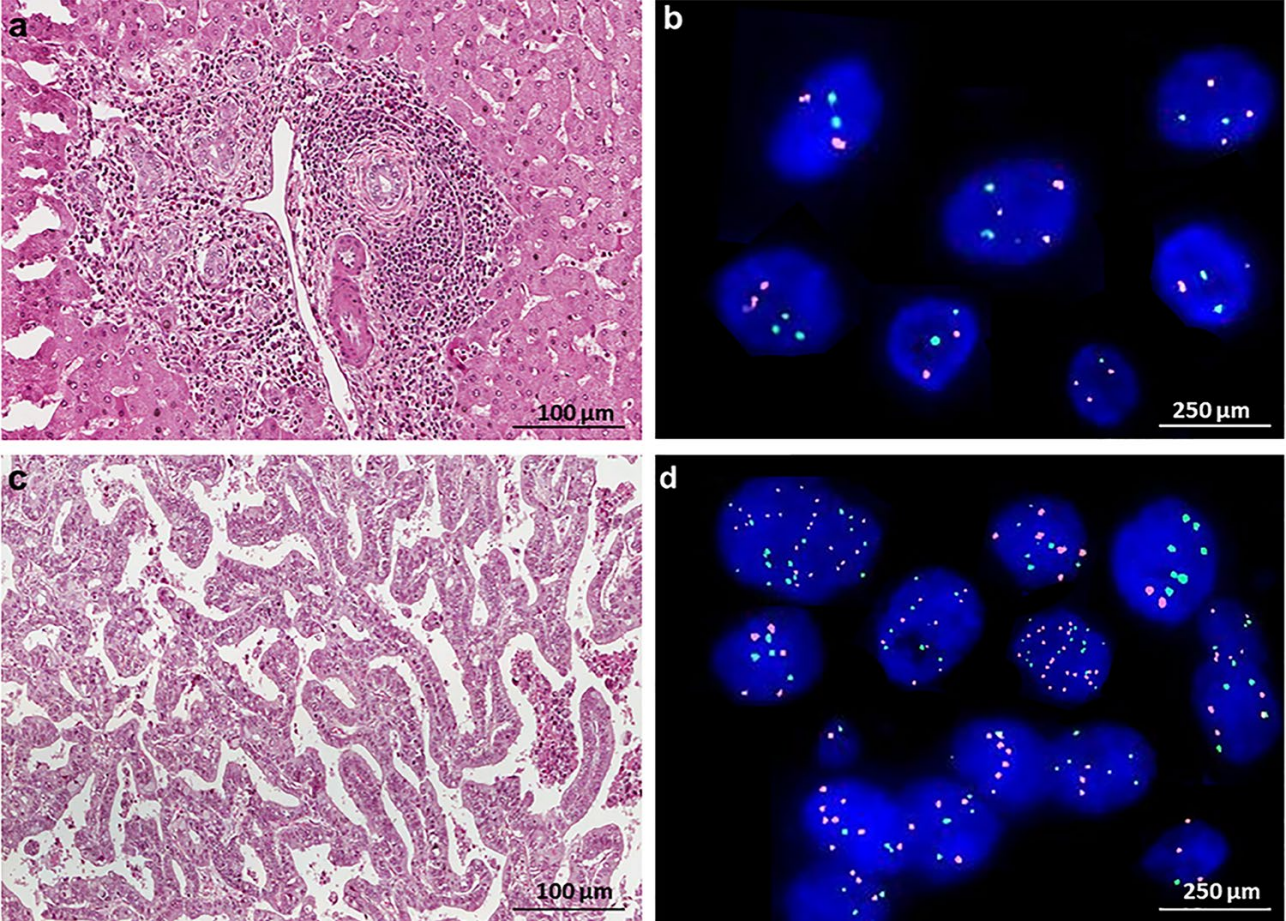
The representative figures of chromosomal aberrations in CCA and cholangitis patients. FFPE from a cholangitis patient; H&E staining, 400X, scale bar = 100 µm (**a**) showed disomy C7 and 17 of FISH assay, 1000X, scale bar = 250 µm (**b**). FFPE from a CCA patients of H&E staining, 400X, scale bar = 100 µm (**c**) showed polysomy of C7 and 17 of FISH assay, 1000X, scale bar = 250 µm (**d**).

A total of 157 CCA patients, 77 (49.1%) females and 80 (50.9%) males, were studied in this retrospective study. The age < 60 years old was 87 cases (55.3%) and ≥ 60 years old was 70 cases (46.7%). The clinical data included the levels of AST; 50 cases with < 40 IU (45.5%) and 69 cases with ≥ 40 IU (54.5%), ALT; 54 cases with < 40 IU (45.7%) and 64 cases with ≥ 40 IU (54.3%) and ALP; 42 cases with < 130 IU (35.6%) and 76 cases with ≥ 130 IU (64.4%). Based on the tumor size, 97 cases (61.1%) have small tumor (≤ 5 cm) and 61 cases (38.9%) has large one (> 5 cm). By the anatomical location, intrahepatic CCA was 63 cases (40.1%) and extrahepatic CCA was 94 cases (57.9%). According to gross pathology, intraductal growth type (ID) was 31 cases (20%), periductal infiltrating type (PI) 45 cases (29%), mass-forming type (MF) 34 cases (22%), and mixed type 45 cases (29%). Histologically, non-papillary type was seen in 75 patients (47.8%) and papillary type was seen in 82 patients (52.2%). Metastases were also characterized. Among 157 patients, 27 cases (17.2%) had gall bladder metastasis, 79 cases (50.3%) had lymph node metastasis and 40 cases (25.5%) had distant metastasis (Tables 1 and 2). Either of increased C7 or C17 copy numbers were investigated in all 157 CCA cases according to the percentage of polysomy cells higher mean cut-off value that calculated from 10 cholangitis patients (> 4.5% and 4% for C7 and C17, respectively). Representative FISH images are shown in Fig. 2.

**Table 1:** The correlation of clinicopathological characteristics of CCA patients with chromosome 7 copy number variants.

Variables	Number	CNV of 7, n (%)			*P*-value
		Disomy	Trisomy	> Trisomy	
**Age (year)**					
< 60	87	29 (60.4%)	22 (51.2%)	36 (54.6.%)	0.726
≥ 60	70	19 (39.6%)	21 (48.8%)	30 (45.5%)	
**Gender**					
Male	80	25 (52.1%)	27 (62.8%)	28(42.4%)	0.093
Female	77	23 (47.9%)	16 (37.2%)	38(57.6%)	
**AST**^**a**^					
< 40	50	16 (35.6%)	23 (57.5%)	11(32.4%)	0.050*^†^
≥ 40	69	29 (64.4%)	17 (42.5%)	23(67.6%)	
**ALT**					
< 40	54	18 (40.0%)	23 (57.5%)	13 (39.4%)	0.186
≥ 40	64	27 (60.0%)	17 (42.5%)	20 (60.6%)	
**ALP**					
< 130	42	19(42.2%)	12 (30.8%)	11 (32.4%)	0.493
≥ 130	76	26 (57.8%)	27 (69.2%)	23 (67.6%)	
**Tumor size (cm)**					
Small (≤ 5 cm)	96	36 (72%)	28 (65.1%)	34 (51.5%	0.085
Large (> 5 cm)	61	14 (28%)	15 (34.9%)	32 (48.5%)	
**Anatomical position**					
Intrahepatic	63	22 (45.8%)	16 (37.2%)	25 (37.9%)	0.840
Extrahepatic	94	26 (54.2%)	27 (62.8%)	41 (62.1%)	
**Gross pathology**^**b**^					
ID	31	15 (31.3%)	8 (19.5%)	8 (12.1%)	**0.036**^**#**^
PI	45	16 (33.3%)	11 (26.8%)	18 (27.3%)	
MF	34	5 (10.4%)	7 (17.1%)	22 (33.3%)	
Mixed types	45	12 (25.0%)	15 (36.6%)	18 (27.3%)	
**Histological type**					
Non-Papillary	75	21 (43.8%)	21 (50.0%)	33 (47.1%)	0.804
Papillary	82	27 (56.3%)	21 (50.0%)	37 (52.9%)	
**Lymph node metastasis**^**c**^					
No	78	37 (75.5%)	20 (46.5%)	21 (32.3%)	< 0.001*^#^
Yes	79	12 (24.5%)	23 (53.5%)	44 (67.7%)	
**Gall bladder metastasis**^**d**^					
No	130	45 (95.7%)	39 (90.7%)	46 (69.7%)	**0.001**^**#†**^
Yes	27	3 (4.3%)	4 (9.3%)	20 (30.3%)	
**Distant metastasis**^**e**^					
No	117	46 (95.8%)	29 (67.4%)	42 (63.6%)	< 0.001*^#^
Yes	40	2 (4.2%)	14 (32.6%)	24 (36.4%)	

*P < 0.05 Disomy vs Trisomy; ^#^P < 0.05 Disomy vs > Trisomy; ^†^P < 0.05 Trisomy vs > Trisomy.

^a^AST: Disomy vs Trisomy; P = 0.043, Trisomy vs > Trisomy; P = 0.031.

^b^Gross pathology: Disomy vs > Trisomy; P = 0.009.

^c^Lymnode Metastasis: Disomy vs Trisomy; P = 0.004, Disomy vs > Trisomy; P < 0.001.

^d^Gall bladder Metastasis: Disomy vs > Trisomy; P = 0.001, vs > Trisomy; P = 0.009.

^e^Distant Metastasis: Disomy vs Trisomy; P < 0.001, Disomy vs > Trisomy; P < 0.001.

**Table 2:** The correlation of C17 CNVs with clinicopathological features of CCA patients.

Variables	No	CNV of C17, n (%)			*P*-value
		Disomy	Trisomy	> Trisomy	
**Age (year)**					
< 60	87	33 (62.3%)	9 (45.0%)	45 (53.6%)	0.341
≥ 60	70	20 (37.7%)	11 (55.0%)	39 (46.4%)	
**Gender**					
Male	80	26 (49.1%)	15 (75.0%)	39 (46.4%)	0.063
Female	77	27 (50.9%)	5 (25.0%)	45 (53.6%)	
**AST**^**a**^					
< 40	50	18 (36.0%)	13 (68.4%)	19 (38%)	0.032*^†^
≥ 40	69	32 (64.0%)	6 (31.6%)	31 (62%)	
**ALT**					
< 40	54	19 (38.0%)	12 (63.2%)	23 (46.9%)	0.191
≥ 40	64	31 (62.0%)	7 (36.8%)	26 (53.1%)	
**ALP**					
< 130	42	20 (40.0%)	4 (22.2%)	18 (36%)	0.547
≥ 130	76	30 (60.0%)	14 (77.8%)	32 (64%	
**Tumor size (cm)**					
Small (≤ 5 cm)	96	37 (69.8%)	12 (60.0%)	47 (56%)	0.210
Large (> 5 cm)	61	16 (30.2%)	8 (40.0%)	37 (44%)	
**Anatomical position**					
Intrahepatic	63	24 (45.3%)	7 (35.0%)	32 (38.1%)	0.844
Extrahepatic	94	29 (54.7%)	13 (65.0%)	52 (61.9%)	
**Gross pathology**^**b**^					
ID	31	17 (32.1%)	4 (21.1%)	10 (12%)	**0.023**^**#**^
PI	45	17 (32.1%)	6 (31.6%)	22 (26.5%)	
MF	34	6 (11.3%)	2 (10.5%)	26 (31.3%)	
Mixed types	45	13 (24.5%)	7 (36.8%)	25 (30.2%)	
**Histologic type**					
Non-Papillary	75	24 (45.3%)	9 (47.4%)	42 (49.4%)	0.885
Papillary	82	29 (54.7%)	10 (52.6%)	43 (50.6%)	
**Lymph node metastasis**^**c**^					
No	78	40 (74.1%)	9 (45.0%)	29 (34.9%)	< 0.001*^#^
Yes	79	14 (25.9%)	11 (55.0%)	54 (65.1%)	
**Gall bladder metastasis **^**d**^					
No	130	50 (94.3%)	19 (95.0%)	61 (72.6%)	** < 0.001**^**#**†^
Yes	27	3 (5.7%)	1 (5.0%)	23 (27.4%)	
**Distant metastasis**^**e**^					
No	117	51 (96.2%)	16 (80.0%)	50 (59.5%)	< 0.001*^#^
Yes	40	2 (3.8%)	4 (20.0%)	34 (40.5%)	

*P < 0.05 Disomy vs Trisomy; ^#^ P < 0.05 Disomy vs > Trisomy; ^†^P < 0.05 Trisomy vs > Trisomy.

^a^AST Disomy vs Trisomy; P = 0.016, Trisomy vs > Trisomy;P = 0.019.

^b^Gross Pathology: Disomy vs > Trisomy; P = 0.006.

^c^Lymnode Metastasis: Disomy vs Trisomy; P = 0.019;Disomy vs > Trisomy; P < 0.001.

^d^Gall bladder Metastasis: Disomy vs > Trisomy; P = 0.002, Trisomy vs > Trisomy y; P = 0.040.

^e^Distant Metastasis: Disomy vs Trisomy; P = 0.023, Disomy vs > Trisomy; P = 0.001.

### The correlation between C7 and C17 CNVs and clinicopathological features of CCA patients

**Figure 3:**
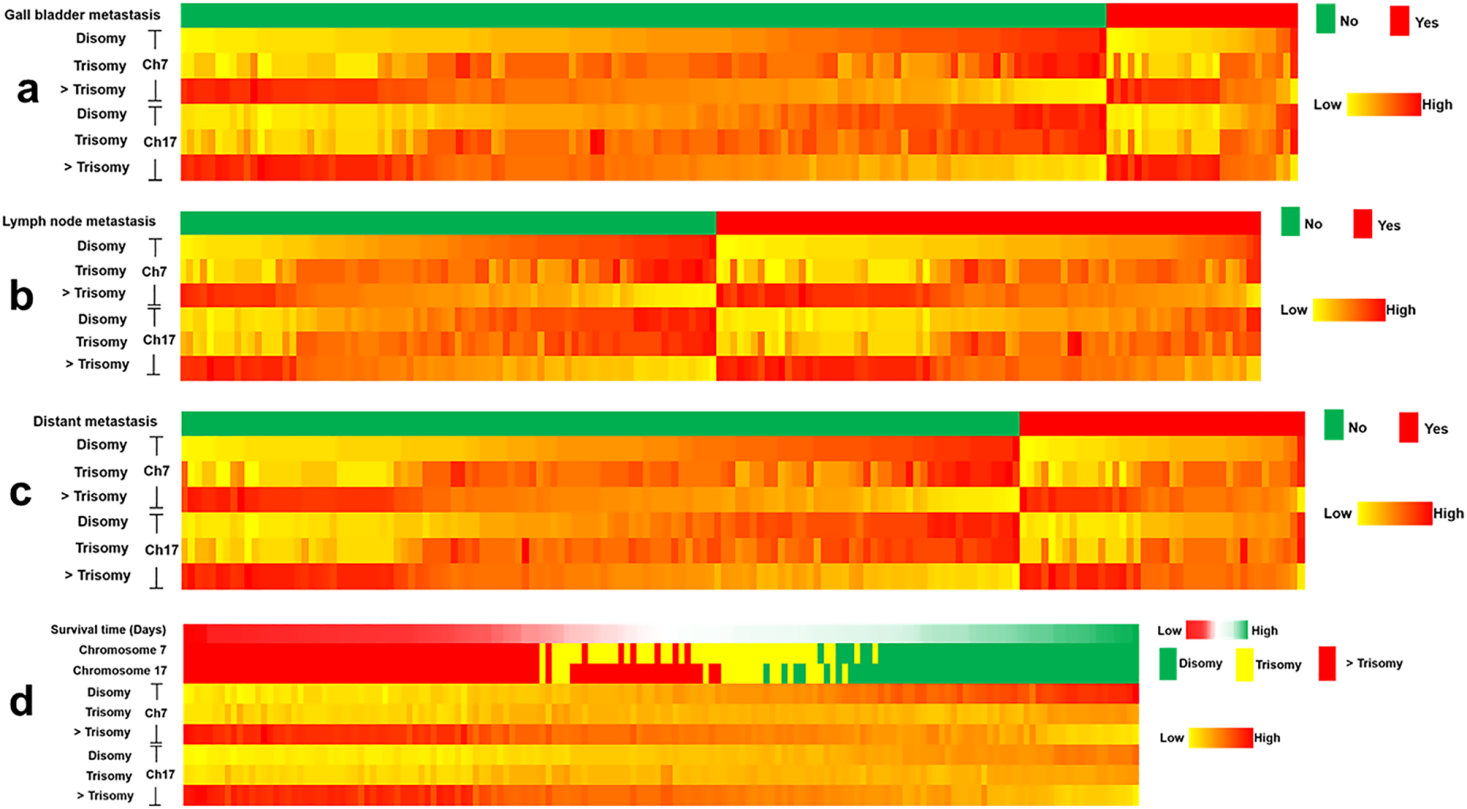
Heatmap analysis showing the proportion of C7 and 17 disomy and polysomy in patients with gall bladder metastasis (**a**), lymph node metastasis (**b**), distant metastasis (**c**) and survival time (**d**).

As shown in Fig. 1, the chromosome phenotypes were defined as disomy, trisomy, and more than trisomy using FISH technique. The correlation between distribution patterns of C7 copy number and clinical features of CCA patients were examined and the results were summarized in Table 1. The incidence of C7 copy number variants (CNVs) was significantly higher in patients with higher AST level (*P* = 0.05). The difference of the incidence of CNVs in between disomy and trisomy group was as same as that in between trisomy vs. > trisomy (*P* = 0.031). In addition, the incidence of CNVs was significantly associated with the gross pathological types particularly). Disomy was significant difference from > trisomy. Patients who had gall bladder, lymph node and distant metastasis have greater proportion of both C7 and C17 > trisomy than those patients who had no evidence of gall bladder metastasis (Fig. 3a–c). Moreover, the prevalence of C7 CNVs significantly correlated with distant metastasis (*P* < 0.001) (Table 1). The incidence of C17 CNVs was significantly higher in patients with high AST level. There were significant differences in the incidence of disomy and trisomy as well as trisomy vs. > trisomy. The incidence of C17 CNVs was significantly associated with the gross pathologic types. The incidence of C17 CNVs also significantly correlated with gall bladder, lymph node and distant metastasis (*P* < 0.001) (Table 2).

### The correlation between clinicopathological factors, C7/C17 CNVs and metastatic features

Univariate and multivariate analyses were applied to evaluate the correlations between FISH results of C7/C17 CNVs and metastatic features including lymph node, gall bladder and distant metastasis and other clinicopathological factors including demographic data, some liver function data, histopathology in CCA patients (Tables 3 and 4).

**Table 3:** Univariate analysis of the clinicopathological factors and metastasis.

Variables	No	Lymph node metastasis		GB metastasis		Distant metastasis	
		OR (95%CI)	*P*-value	OR (95%CI)	*P*-value	OR (95%CI)	*P*-value
**Age (year)**							
< 60	87	1		1		1	
≥ 60	70	0.646 (0.343–1.217)	0.176	0.566 (0.237–1.352)	0.200	1.534 (0.746–3.155)	0.245
**Gender**							
Male	81	1		1		1	
Female	77	0.951 (0.509–1.774)	0.874	0.972 (0.424–2.227)	0.947	1.224 (0.597–2.509)	0.582
**AST**							
< 40	50	1		1		1	
≥ 40	69	0.971 (0.469–2.012)	0.938	1.702 (0.599–4.840)	0.318	0.956 (0.406–2.251)	0.918
**ALT**							
< 40	54	1		1		1	
≥ 40	64	0.714 (0.345–1.478)	0.364	1.193 (0.442–3.220)	0.727	0.548 (0.233–1.292)	0.169
**ALP**							
< 130	42	1		1		1	
≥ 130	76	1.562 (0.731–3.338)	0.250	3.467 (0.947–12.686)	0.060	01.417 (0.560–3.586)	0.462
**Tumor size (cm)**							
Small (≤ 5 cm)	97	1		1		1	
Large (> 5 cm)	61	1.307 (0.687–2.484)	0.414	0.923 (0.392–2.173)	0.854	2.155 (1.040–4.465)	0.039
**Anatomical position**							
Intrahepatic	63	1		1		1	
Extrahepatic	95	3.524 (1.797–6.908)	** < 0.001**	4.712 (1.543–14.386)	**0.006**	0.864 (0.417–1.790)	0.695
**Gross pathology**							
ID	31	1		1		1	
PI	45	5.702 (1.957–16.614)	**0.001**	7.778 (0.898–67.364)	0.063	1.038 (0.267–4.034)	0.957
MF	34	4.687 (1.534–14.322)	**0.007**	9.706 (1.182–79.670)	**0.034**	7.594 (2.181–26.437)	0.001
Mixed types	45	5.702 (1.957–16.614)	**0.001**	5.526 (0.644–47.408)	0.119	2.455 (0.710–8.487)	0.156
**Histology type**							
Non-papillary	75	1		1		1	
Papillary	82	1.082 (0.576–2.032)	0.807	0.745 (0.320–1.735)	0.495	0.473 (0.225–0.995)	**0.048**
**C7**							
Disomy	49	1		1		1	
Trisomy	43	3.546 (1.464–8.591)	**0.005**	1.573 (0.332–7.458)	0.569	11.345 (2.403–53.568)	**0.002**
> Trisomy	66	6.027 (2.629–13.812)	** < 0.001**	6.815 (1.893–24.539)	**0.003**	13.756 (3.063–61.778)	**0.001**
**C17**							
Disomy	54	1		1		1	
Trisomy	20	3.492 (1.197–10.188)	**0.022**	0.895 (0.088–9.138)	0.925	6.500 (1.088–38.833)	**0.040**
> Trisomy	84	5.048 (2.371–10.746)	** < 0.001**	6.131 (1.735–21.665)	**0.005**	17.160 (3.910–75.318)	** < 0.001**
**C7/17**							
Disomy	47	1		1		1	
Trisomy	18	4.583 (1.448–14.509)	**0.010**	0.863 (0.084–8.879)	0.901	9.200 (0.889–95.221)	0.063
> Trisomy	60	5.417 (2.331–12.590)	** < 0.001**	5.333 (1.451–19.609)	**0.012**	24.769 (3.186–192.588)	**0.002**

**Table 4:** Multivariate analysis of the factors related to metastasis.

Variables	Multivariate (backward)		
	OR	95% CI	*P*-value
**Lymph node metastasis**			
Anatomical position (intrahepatic/extrahepatic)	**3.249**	1.412–7.478	**0.006**
Gross pathology (ID/PI)	3.427	1.000–11.749	**0.050**
Gross pathology (ID /MF)	2.884	0.834–9.974	0.094
Gross pathology (ID/mixed)	3.174	0.948–10.630	0.061
C7 (disomy/trisomy)	**3.496**	1.306–9.359	**0.013**
C7 (disomy/ > trisomy)	**6.944**	2.695–17.896	** < 0.001**
C17 (disomy/trisomy)	1.707	0.348–8.386	0.510
C17 (disomy/ > trisomy)	1.478	0.253–8.642	0.665
C7/17 (disomy/trisomy)	**4.488**	1.284–15.682	**0.019**
C7/17 (disomy/ > trisomy)	**6.723**	2.663–16.973	** < 0.001**
**GB metastasis**			
Anatomical position (intrahepatic/extrahepatic)	**4.473**	1.398–14.309	**0.012**
Gross pathology (ID/PI)	0.745	0.178–3.119	0.687
Gross pathology (ID /MF)	4.763	1.252–18.120	**0.022**
Gross pathology (ID/mixed)	1.637	0.437–6.134	0.465
C7 (disomy/trisomy)	–	–	–
C7 (disomy/ > trisomy)	12.722	2.848–56.824	**0.001**
C17 (disomy/trisomy)	1.215	0.102–14.465	0.877
C17 (disomy/ > trisomy)	**9.515**	2.098–43.148	**0.003**
C7/17 (disomy/trisomy)	1.209	0.101–14.512	0.881
C7/17 (disomy/ > trisomy)	**8.000**	1.707–37.486	**0.008**
**Distant metastasis**			
Anatomical position (intrahepatic/extrahepatic)	–	–	–
Gross pathology (ID/PI)	1.059	0.164–6.815	0.952
Gross pathology (ID/MF)	5.921	1.055–33.224	0.043
Gross pathology (ID/mixed)	1.457	0.238–8.927	0.684
C7 (disomy/trisomy)	1.142	0.069–18.858	0.926
C7 (disomy/ > trisomy)	0.358	0.017–7.572	0.509
C17 (disomy/trisomy)	6.576	0.397–108.876	0.188
C17 (disomy/ > trisomy)	31.130	1.609–602.161	**0.023**
C7/17 (disomy/trisomy)	10.037	0.922–109.229	0.058
C7/17 (disomy/ > trisomy )	17.076	2.119–137.615	**0.008**

In the univariate analysis, anatomical position; extrahepatic CCA, gross pathology; periductal infiltrating type (PI), mass forming type (MF), mixed type, FISH results; C7 trisomy, C7 > trisomy, C17 trisomy, C17 > trisomy, C7 and C17 trisomy, C7 and C17 > trisomy were significantly associated with lymph node metastasis (P < 0.05). Gall bladder metastasis was significantly associated with eCCA, PI, C7 > trisomy, C17 > trisomy, C7 and C17 > trisomy. Moreover, tumor size large > 5 cm, MF, histological type; non-papillary, C7 trisomy, C7 > trisomy, C17 trisomy, C17 polysomy, C7 and C17 > trisomy were significantly correlated with distant metastasis (*P* < 0.05) (Table 3).

In the multivariate (backward) analyses, eCCA (OR, 3.249, 95% CI 1.412–7.478; *P* = 0.006), C7 trisomy (OR, 3.496, 95% CI 1.306–9.359; *P* = 0.013), C7 > trisomy (OR, 6.944, 95% CI 2.695–17.896; *P* < 0.001), C7/17 trisomy (OR, 4.488, 95% CI 1.284–15.682; *P* = 0.019), and C7/17 > trisomy (OR, 6.723, 95% CI 2.663–16.973; *P* < 0.001) were independent predictive factors for lymph node metastasis. For gall bladder metastasis, the factors including eCCA (OR, 4.473, 95% CI 1.398–14.309; *P* = 0.012), C17 > trisomy (OR, 9.515, 95% CI 2.098–43.148; *P* = 0.003), and C7/17 (OR, 8, 95% CI 1.707–37.486; *P* = 0.008) were also independent predictive factors. Moreover, mass forming gross type (OR, 5.921, 95% CI 1.055–33.224; *P* = 0.043), C7/17 > trisomy (OR, 17.076, 95% CI 2.119–137.615; *P* = 0.008) and particularly, C17 (OR, 31.130, 95% CI 1.609–602.161; *P* = 0.023), were independent predictive factors of organ metastasis.

### The clinicopathological parameters and chromosome aberrations to predict overall survival time

**Figure 4:**
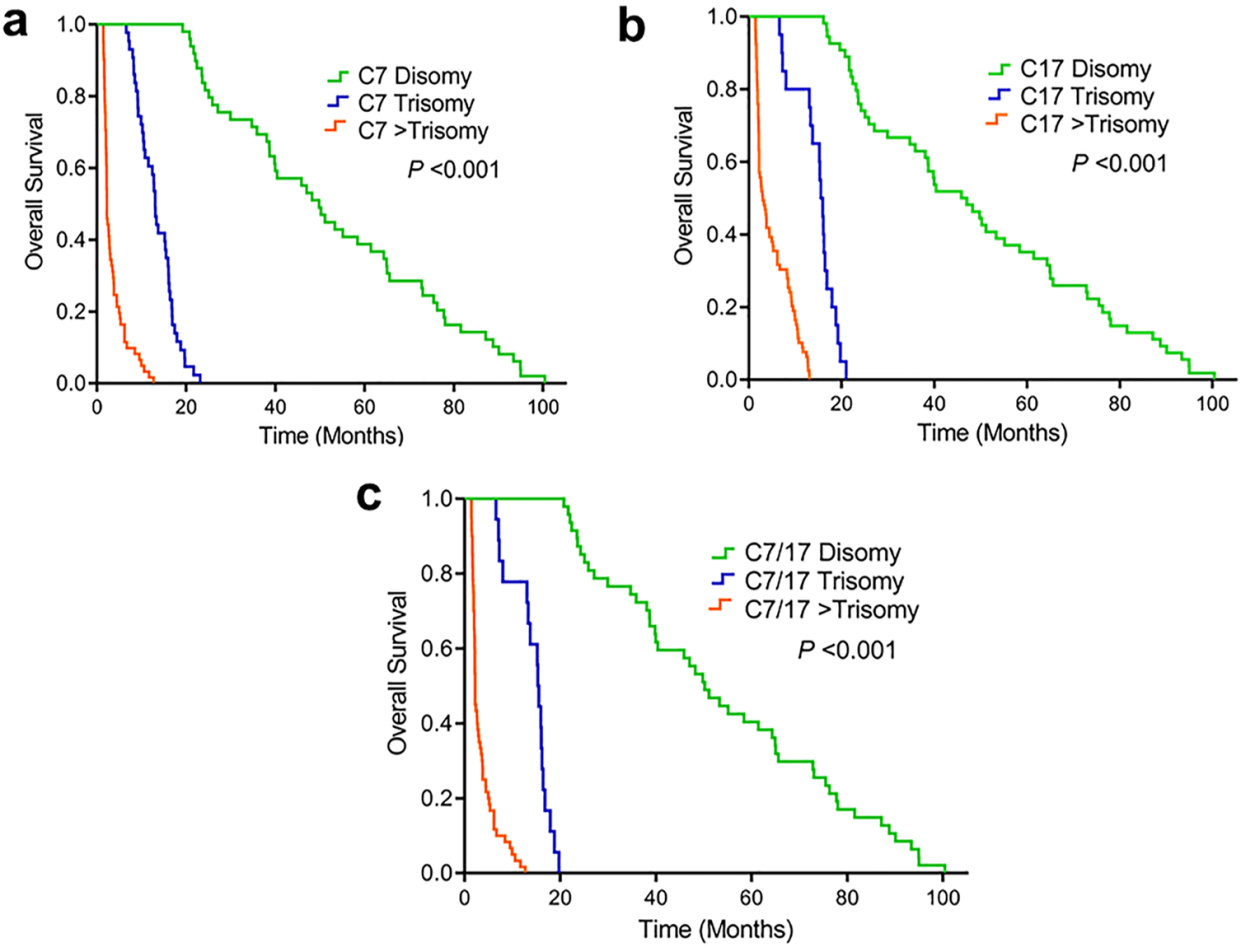
Kaplan–Meier analysis for overall survival time with an increased chromosome 7, 17 and both 7/17 copy number in CCA patients (**a**–**c**).

The association between overall survival time (OS) and clinicopathological parameters or chromosome aberrations was evaluated using univariate analysis (Table 5) and Kaplan–Meier analysis, and the curves were compared using log-rank tests (Fig. 4). In this analyses, AST ≤ 40, lymph node metastasis, gall bladder metastasis, distant metastasis, mass forming gross type, C7 trisomy, C7 > trisomy, C17 trisomy, C17 > trisomy, C7/17 trisomy and C7/17 > trisomy (p < 0.001) were significantly associated with OS. The median OSs of the patients having normal AST and high AST level were 15.97 months and 16.13 months, respectively. The median OSs of the patients with and without lymph node metastasis were 7.2 months and 19.72 months. In terms of gall bladder metastasis, median OS of the patients with and without gall bladder metastasis was 2.73 months vs. 13.22 months. Similarly, the median OS of the patients with and without distant metastasis were 7 months and 16.18 months, respectively. Heat map analysis show that among overall CCA cases, patients who had longer survival time showed higher > trisomy ratio of both C7 and C17 than those with shorter survival time (Fig. 3d). In multivariate analyses, lymph node metastasis (hazard ratio, 1.923; 95% CI 1.204–3.072; *P* = 0.006), C7 trisomy (HR, 24.455; 95% CI 7.202–83.044; *P* < 0.001), C7 > trisomy (HR, 80.783; 95% CI 20.288–321.657; *P* < 0.001), C17 trisomy (HR, 7.169; 95% CI 2.301–22.339; *P* = 0.001) and C17 > trisomy (hazard ratio, 61.665; 95% CI 13.194–288.199; *P* < 0.001), C7/17 trisomy (HR, 28.966; 95% CI 2.796–42.420; *P* < 0.001), in addition C7/17 > trisomy (HR, 395.870; 95% CI 18.450–433.42; *P* < 0.001) were independent predictive factors of poor OS (Table 6). In consideration of C7 aberrations in CCA patients, the OS of the patients with trisomy and > trisomy was significantly shorter than that with normal C7 (log rank *P* < 0.001; median OS 2.23 vs.13.07 vs.49.77 months), respectively (Fig. 4a). Likewise, the patients with > trisomy C17 and trisomy C17 had shorter OS than those patients with normal C17 (log rank *P* < 0.001; median OS 3.03 vs.15.53 vs.45.83 months) (Fig. 4b). Moreover, the patients with > trisomy of C7/17 and trisomy of C7/17 had shorter survival time than those patients with normal C7/17 (log rank *P* < 0.001; median OS 2.23 vs.15.33 vs. 50.23 months), respectively (Fig. 4c).

**Table 5:** Univariate of clinicopathological parameters, chromosomal aberrations and overall survival time.

Variables	Number	Median OS (months)	Univariate	
			HR (95%CI)	*P*-value
**Age (year)**				
< 60	84	12.75	1	
≥ 60	69	9.97	1.157 (0.839–1.596)	0.374
**Gender**				
Male	80	12.94	1	
Female	74	8.99	0.852 (0.613–1.183)	0.338
**AST**				
< 40	49	15.97	1	
≥ 40	67	16.13	0.664 (0.447–0.987)	**0.043**
**ALT**				
< 40	52	14.48	1	
≥ 40	63	17.43	0.763 (0.525–1.111)	0.158
**ALP**				
< 130	42	16.92	1	
≥ 130	73	15.53	1.161 (0.791–1.705)	0.445
**Tumor size (cm)**				
Small (≤ 5 cm)	95	13.73	1	
Large (> 5 cm)	59	9.17	1.385 (0.996–1.926)	0.053
**Lymph node metastasis**				
No	78	19.72	1	
Yes	76	7.20	2.498 (1.772–3.520)	** < 0.001**
**Gall bladder metastasis**				
No	130	13.22	1	
Yes	24	2.73	1.809 (1.159–2.823)	**0.009**
**Distant metastasis**				
No	116	16.18	1	
Yes	38	7.00	2.104 (1.427–3.102)	** < 0.001**
**Anatomical position**				
Intrahepatic	63	12.70	1	
Perihilar Extrahepatic	83	11.53	1.100 (0.789–1.532)	0.574
Distal Extrahepatic	8	16.25	1.098 (0.525–2.296)	0.804
**Anatomical position**				
Intrahepatic	63	12.70	1	
Extrahepatic	91	11.53	1.100 (0.795–1.521)	0.567
**Gross pathology**				
ID	30	24.54	1	
PI	43	15.33	1.367 (0.852–2.194)	0.195
MF	34	5.67	2.473 (1.492–4.099)	** < 0.001**
Mixed types	44	11.03	1.572 (0.983–2.513)	0.059
**Histology type**				
Non-papillary	75	10.32	1	
Papillary	82	12.70	0.763 (0.548–1.062)	0.109
**C7**				
Disomy	49	49.77	1	
Trisomy	43	13.07	4.723 (2.405–9.275)	** < 0.001**
> Trisomy	62	2.23	112.113 (48.080–261.425)	** < 0.001**
**C17**				
Disomy	54	45.83	1	
Trisomy	20	15.53	29.862 (11.247–79.291)	** < 0.001**
> Trisomy	80	3.03	495.067 (134.308–1824.839)	** < 0.001**
**C7/17**				
Disomy	47	50.23	1	
Trisomy	18	15.33	31.864 (12.257–89.391)	** < 0.001**
> Trisomy	60	2.23	253.020 (27.124–2360.244)	** < 0.001**

**Table 6:** Multivariate of clinicopathological parameters, chromosome aberrations and overall survival time.

Variables	Multivariate		
	HR	95% CI	*P*-value
**Model A**			
AST (< 40/ ≥ 40)	1.090	0.701–1.694	0.703
Lymph node metastasis (No/Yes)	1.923	1.204–3.072	**0.006**
Gall bladder metastasis (No/Yes)	0.856	0.469–1.563	0.613
Distant metastasis (No/Yes)	0.841	0.494–1.432	0.525
C7 (Disomy/Trisomy)	24.455	7.202–83.044	** < 0.001**
C7 (Disomy/ > Trisomy)	80.783	20.288–321.657	** < 0.001**
C17 (Disomy/Trisomy)	7.169	2.301–22.339	**0.001**
C17 (Disomy/ > Trisomy)	61.665	13.194–288.199	** < 0.001**
**Model B**			
AST (< 40/ ≥ 40)	0.897	0.540–1.490	0.676
Lymph node metastasis (No/Yes)	1.943	1.148–3.288	**0.013**
Gall bladder metastasis (No/Yes)	1.017	0.522–1.981	0.960
Distant metastasis (No/Yes)	0.727	0.368–1.440	0.361
C7/17 (Disomy/Trisomy)	28.966	2.796–42.420	** < 0.001**
C7/17 (Disomy/ > Trisomy)	395.870	18.450–433.42	** < 0.001**

## Discussion

CCA is asymptomatic in the early stage and is usually diagnosed at the advanced stage resulting in unsatisfied outcome with poor prognosis in spite of various treatments^9^. Prognostication is important in clinical and ethical approaches of clinicians engaged in oncology and palliative care of CCA, because it helps clinicians for planning an appropriate therapeutic strategy for advanced cancer patients^17^. Prognostication is made mostly based on tumor staging^18,19^. In addition, several factors including chromosomal aberration^21,22^ have been reported as prognostic factors for advanced cancer patients^20^. FISH assay is applied for several cancers including numerical and structural chromosome abnormalities in CCA patients^31^. In this study, we found that an increase of C7 CNVs is associated with high AST level, gross mass forming type and metastasis to lymph node, gall bladder and distant organs. These results indicate that metastatic status that reported potential pathological factors for predicting CCA recurrence^39^ is highly relevant to C7 > trisomy in CCA patients. Furthermore, an increase of C17 CNVs is associated with the clinical parameters such as high AST level, mass forming type, lymph node, gall bladder and distant metastasis. Interestingly, an increase of C7 and C17 CNVs is associated with microscopic growth patterns particularly, mass-forming type which have the poorest prognosis in iCCA patients^40,41^ as same as the metastatic features. AST is useful for the evaluation of bile duct and liver injury that shown a modest impact on the OS of the iCCA patients^42,43^. Thus, in case of poor prognostic iCCA that underwent C7 and C17 aberrations shown associated significantly with high AST level. However, For the mechanisms of association with high AST level and an increase of C7 and C17 CNVs, not yet elucidated.

In the univariate analyses, C7 > trisomy C17 > trisomy and mix C7/17 > trisomy were significantly associated with each metastasis type, to lymph node to gall bladder and to distant organ, respectively. However, in the multivariate analyses, an increase of C7 and C7/17 > trisomy was an independent predictive factor of lymph node metastasis only. Nevertheless, an increase of C17 CNV and C7/17 > trisomy were independent predictive factors of gall bladder metastasis. For distant metastasis, an increase of C17 and C7/17 CNVs were independent predictive factors. Thus, an increase of C7 and C17 strongly correlated with lymph node and distant metastasis that indicated advanced stage CCA followed by TMN and American Joint Committee on Cancer (AJCC)/Union for International Cancer Control (UICC) Staging Systems^44^.

Additionally, in univariate analysis, we found that patients with C7 > trisomy and trisomy have short median survival time than those C7 disomy. Similarly, patients with C17 > trisomy and trisomy have shorter median survival time than those with C17 disomy (OS 3.03 Vs.15.53 Vs.45.83). Moreover, pattientts having both C7 and 17 C7 > trisomy and trisomy showed short median survival time than those with disomy (OS 2.23 Vs.15.33 Vs.50.23), respectively. Thus, an increase of C7, C17 and C7/17 CNVs were significantly associated with poor prognosis in CCA patients in univariate analysis. Interestingly, an increase of C7, C17 copy numbers as trisomy and greater than trisomy were independently correlated with short median survival time by multivariate analysis. Similar to our results, Kato et al. (2008) reported that, using multivariate analysis, an increase of C7 copy number and advanced clinical stage were independently predictive of poor OS^32^. While trisomy C7 is usually associated with biliary tree inflammation, tetrasomy can be seen during the mitotic M phase of biliary cancer^45^. According to previous studies, C7 contains several oncogenes; epidermal growth factor receptor (EGFR) (7p12) and Met (7q31) may play important roles in the development of biliary tract carcinogenesis and/or tumor progression. EGFR overexpression in cancer tissue samples by immunohistochemistry is significantly associated with the clinicopathological features of CCA and is an independent prognostic factor for poor OS^46,47^. Some study found that a novel deletion of MUC17 at 7q22.1 affected prognosis of biliary tract cancer patients which have negative effects on mutated genes including TP53, KRAS, SMAD4, NF1, ARID1A, PBRM1, and ATR^30^. In addition, human epidermal growth factor receptor 2 proto-oncogene (HER-2, also known as c-erbB2, ERBB2) that regulates cell growth, survival, differentiation and migration is located on C17 (q12–q21)^48^. ERBB2 is also the most common genetic alterations in invasive breast carcinomas, associated with poor prognosis and response of the tumor to the ERBB2 monoclonal antibody trastuzumab in breast cancer^49^. Furthermore, ERBB receptors are frequently overexpressed in CCA leading to tumor progression and poor prognosis^50^. Besides, copy number analysis also detected more frequent ERBB2 amplification in liver fluke-related CCAs, which may have considerable clinical implications^40^. ERBB-2, EGFR and Met are members of tyrosine kinase growth factor receptors (TKGFRs) that play major roles in bile duct carcinogenesis. Thereby, overexpression of these genes as mentioned above resulting from an increase of C7 and C17 CNVs elicit an essential role in CCA progression^37,47^. Markedly, an increase of C7, C17 and C7/C17 copy numbers are strongly independent markers for the short survival time of CCA patients. Our study was performed on a large scale CCA specimens over 5 years retrospective research. Therefore, the results of FISH assay using FFPE samples significantly support its potential as prognostic marker of poor prognosis in CCA patient. In further study, we would add more probes or apply UroVysion^®^ probes in extended retrospective duration time.

## Conclusion

An increase of C7, 17 and 7/17 CNVs in FFPE specimens were significant correlated with metastatic factors in CCA patients. In particular, having more than trisomy of C7 and 7/17 were statistically significant independent predictive factor of lymph node metastasis. Also, Patients who have more than trisomy of C17 and 7/17 were statistically significant independent predictive factor of gall bladder metastasis. Moreover, > trisomy of C7/17 was an independent predictive factor of organ metastasis. Patients having trisomy or > trisomy of either C7 or C17 have shorter median survival time than those having normal disomy. Thus, polysomy of both C7 and C17 have a potential as a poor prognostic marker of CCA patients.

## Data Availability

All data sets used and /or analyzed during the study are not publicly available due to personal or identifying data of patients but are available from the corresponding author on reasonable request.

## Abbreviations

CCA: Cholangiocarcinoma
FISH: Fluorescence in situ hybridization
FFPE: Formalin-fixed paraffin-embedded tissues
CEP: Chromosome enumeration probe
iCCA: Intrahepatic cholangiocarcinoma
eCCA: Extrahepatic cholangiocarcinoma
MF: Mass forming type
PI: Periductal infiltrating type
ID: Intraductal growing type

## References

[1] Banales JM (2020). Cholangiocarcinoma 2020: The next horizon in mechanisms and management. Nat. Rev. Gastroenterol. Hepatol..

[2] Sripa B, Pairojkul C (2008). Cholangiocarcinoma: Lessons from Thailand. Curr. Opin. Gastroenterol..

[3] Vatanasapt V (1995). Cancer incidence in Thailand, 1988–1991. Cancer Epidemiol. Biomark. Prev..

[4] Kamsa-Ard S (2019). Cholangiocarcinoma trends, incidence, and relative survival in Khon Kaen, Thailand From 1989 through 2013: A population-based cancer registry study. J. Epidemiol..

[5] Khan SA, Tavolari S, Brandi G (2019). Cholangiocarcinoma: Epidemiology and risk factors. Liver Int..

[6] Khan SA, Toledano MB, Taylor-Robinson SD (2008). Epidemiology, risk factors, and pathogenesis of cholangiocarcinoma. HPB (Oxford).

[7] IARC. A Review of Human Carcinogens: Opisthorchis viverrini and Clonorchis sinensis. IARC Monographs on the Evaluation of Carcinogenic Risks to Humans **100B**, 341-370 (2012) PMC7681589 7715069

[8] Nakanuma Y, Kakuda Y (2015). Pathologic classification of cholangiocarcinoma: New concepts. Best Pract. Res. Clin. Gastroenterol..

[9] Banales JM (2016). Expert consensus document: Cholangiocarcinoma: Current knowledge and future perspectives consensus statement from the European Network for the Study of Cholangiocarcinoma (ENS-CCA). Nat. Rev. Gastroenterol. Hepatol..

[10] Sia D, Villanueva A, Friedman SL, Llovet JM (2017). Liver cancer cell of origin, molecular class, and effects on patient prognosis. Gastroenterology.

[11] Fan B (2012). Cholangiocarcinomas can originate from hepatocytes in mice. J. Clin. Invest..

[12] Wang J (2018). Notch2 controls hepatocyte-derived cholangiocarcinoma formation in mice. Oncogene.

[13] Blechacz B, Komuta M, Roskams T, Gores GJ (2011). Clinical diagnosis and staging of cholangiocarcinoma. Nat. Rev. Gastroenterol. Hepatol..

[14] Krasinskas AM (2018). Cholangiocarcinoma. Surg. Pathol. Clin..

[15] Waraasawapati S, Deenonpoe R, Sa-ngiamwibool P, Chamgramol Y, Pairojkul C, Tabibian JH (2021). Diagnosis and Managementof Cholangiocarcinoma.

[16] Rizvi S, Khan SA, Hallemeier CL, Kelley RK, Gores GJ (2018). Cholangiocarcinoma—evolving concepts and therapeutic strategies. Nat. Rev. Clin. Oncol..

[17] Glare PA, Sinclair CT (2008). Palliative medicine review: Prognostication. J. Palliat Med..

[18] Lamont EB, Christakis NA (2003). Complexities in prognostication in advanced cancer: "to help them live their lives the way they want to". JAMA.

[19] Vigano A, Dorgan M, Buckingham J, Bruera E, Suarez-Almazor ME (2000). Survival prediction in terminal cancer patients: A systematic review of the medical literature. Palliat Med..

[20] Maltoni M (2005). Prognostic factors in advanced cancer patients: evidence-based clinical recommendations—a study by the Steering Committee of the European Association for Palliative Care. J. Clin. Oncol..

[21] Kou F, Wu L, Ren X, Yang L (2020). Chromosome abnormalities: New insights into their clinical significance in cancer. Mol. Ther. Oncolytics.

[22] Albertson DG, Collins C, McCormick F, Gray JW (2003). Chromosome aberrations in solid tumors. Nat. Genet..

[23] Solomon E, Borrow J, Goddard AD (1991). Chromosome aberrations and cancer. Science.

[24] Frohling S, Dohner H (2008). Chromosomal abnormalities in cancer. N. Engl. J. Med..

[25] Homayounfar K (2009). Pattern of chromosomal aberrations in primary liver cancers identified by comparative genomic hybridization. Hum. Pathol..

[26] Koo SH (2001). Genetic alterations in hepatocellular carcinoma and intrahepatic cholangiocarcinoma. Cancer Genet. Cytogenet..

[27] McKay SC (2011). Array comparative genomic hybridization identifies novel potential therapeutic targets in cholangiocarcinoma. HPB (Oxford).

[28] Miller G (2009). Genome wide analysis and clinical correlation of chromosomal and transcriptional mutations in cancers of the biliary tract. J. Exp. Clin. Cancer Res..

[29] Tsuda H, Hirohashi S (1992). Frequent occurrence of p53 gene mutations in uterine cancers at advanced clinical stage and with aggressive histological phenotypes. Jpn. J. Cancer Res..

[30] Wardell CP (2018). Genomic characterization of biliary tract cancers identifies driver genes and predisposing mutations. J. Hepatol..

[31] Ratan ZA (2017). Application of fluorescence in situ hybridization (FISH) technique for the detection of genetic aberration in medical science. Cureus.

[32] Kato A (2018). An increased chromosome 7 copy number in endoscopic bile duct biopsy specimens is predictive of a poor prognosis in cholangiocarcinoma. Dig. Dis. Sci..

[33] DeHaan RD (2007). An assessment of chromosomal alterations detected by fluorescence in situ hybridization and p16 expression in sporadic and primary sclerosing cholangitis-associated cholangiocarcinomas. Hum. Pathol..

[34] Chan-On W (2013). Exome sequencing identifies distinct mutational patterns in liver fluke-related and non-infection-related bile duct cancers. Nat. Genet..

[35] Jusakul A (2017). Whole-genome and epigenomic landscapes of etiologically distinct subtypes of cholangiocarcinoma. Cancer Discov..

[36] Ong CK (2012). Exome sequencing of liver fluke-associated cholangiocarcinoma. Nat. Genet..

[37] Miyamoto M (2011). Prognostic significance of overexpression of c-Met oncoprotein in cholangiocarcinoma. Br. J. Cancer.

[38] Wang H, Wang H, Zhang W, Fuller GN (2002). Tissue microarrays: Applications in neuropathology research, diagnosis, and education. Brain Pathol..

[39] Komaya K (2017). Recurrence after resection with curative intent for distal cholangiocarcinoma. Br. J. Surg..

[40] Brindley PJ (2021). Cholangiocarcinoma. Nat. Rev. Dis. Primers.

[41] Sirica AE (2009). Intrahepatic cholangiocarcinoma progression: Prognostic factors and basic mechanisms. Clin. Gastroenterol. Hepatol..

[42] Kubo S (2016). Screening and surveillance for occupational cholangiocarcinoma in workers exposed to organic solvents. Surg. Today.

[43] Zhang C (2017). Serum liver enzymes serve as prognostic factors in patients with intrahepatic cholangiocarcinoma. Onco Targets Ther..

[44] Blechacz B (2017). Cholangiocarcinoma: Current knowledge and new developments. Gut Liver.

[45] Belchacz, B. in *Sleisenger and Fortran’s Gastrointestinal and Liver Disease* Vol. 1 (ed M Feldman) 1171–1176 (Philadelphia: Saunders, 2010).

[46] Yoshikawa D (2008). Clinicopathological and prognostic significance of EGFR, VEGF, and HER2 expression in cholangiocarcinoma. Br. J. Cancer.

[47] Nakazawa K (2005). Amplification and overexpression of c-erbB-2, epidermal growth factor receptor, and c-met in biliary tract cancers. J. Pathol..

[48] Rubin I, Yarden Y (2001). The basic biology of HER2. Ann. Oncol..

[49] Salido M (2005). Polysomy of chromosome 17 in breast cancer tumors showing an overexpression of ERBB2: a study of 175 cases using fluorescence in situ hybridization and immunohistochemistry. Breast Cancer Res..

[50] Pellat A, Vaquero J, Fouassier L (2018). Role of ErbB/HER family of receptor tyrosine kinases in cholangiocyte biology. Hepatology.

